# Multilevel RTN Removal Tools for Dynamic FBG Strain Measurements Corrupted by Peak-Splitting Artefacts

**DOI:** 10.3390/s22010092

**Published:** 2021-12-23

**Authors:** Dominik Johannes Marius Fallais, Maximilian Henkel, Nymfa Noppe, Wout Weijtjens, Christof Devriendt

**Affiliations:** 1 OWI-Lab, Acoustics and Vibrations Research Group (AVRG), Vrije Universiteit Brussel, Pleinlaan 2, 1050 Brussels, Belgium; maximilian.henkel@vub.be (M.H.); Wout.Weijtjens@vub.be (W.W.); Christof.Devriendt@vub.be (C.D.); 2 24SEA, Drukpersstraat 4, 1000 Brussels, Belgium; Nymfa.Noppe@24sea.be

**Keywords:** peak-splitting, FBG, signal reconstruction, denoising, multi-level random telegraph noise, outlier detection, outlier replacement, operational strain measurements, offshore wind

## Abstract

Strain measurements using fibre Bragg grating (FBG) optical sensors are becoming ever more commonplace. However, in some cases, these measurements can become corrupted by sudden jumps in the signal, which manifest as spikes or step-like offsets in the data. These jumps are caused by a defect in the FBG itself, which is referred to as peak-splitting. The effects of peak splitting artefacts on FBG strain measurements show similarities with an additive multi-level telegraph noise process, in which the amplitudes and occurrences of the jumps are related to fibre deformation states. Whenever it is not possible to re-assess the raw spectral data with advanced peak tracking software, other means for removing the jumps from the data have to be found. The two methods presented in this article are aimed at removing additive multi-level random telegraph noise (RTN) from the raw data. Both methods are based on denoising the sample wise difference signal using a combination of an outlier detection scheme followed by an outlier replacement step. Once the difference signal has been denoised, the cumulative sum is used to arrive back at a strain time series. Two methods will be demonstrated for reconstructing severely corrupted strain time series; the data for this verification has been collected from sub-soil strain measurements obtained from an operational offshore wind-turbine. The results show that the proposed methods can be used effectively to reconstruct the dynamic content of the corrupted strain time series. It has been illustrated that errors in the outlier replacements accumulate and can cause a quasi-static drift. A representative mean value and drift correction are proposed in terms of an optimization problem, which maximizes the overlap between the reconstruction and a subset of the raw data; whereas a high-pass filter is suggested to remove the quasi static drift if only the dynamic band of the signal is of interest.

## 1. Introduction

Strain measurements using optical fibre Bragg grating (FBG) sensors are based on interpreting the reflected spectrum of a light pulse which is sent through an optical fibre; the reflection is caused whenever a light pulse encounters a periodic modulation of the refractive index (grating) etched into the optical fibre. Theoretically, the reflected spectrum is narrowbanded and characterized by a single peak centred at the Bragg-wavelength, which is proportional to the grating period. Deforming the underlying specimen results in an elongation or compression of the grating and therewith in a shift of the wavelength of the reflected spectrum. As a result, the time history of the wavelength will resemble the strain loading of the underlying specimen [1].

However, due to several failure modes, the reflected spectrum can be deformed, potentially resulting in two or more peaks of comparable amplitudes, which is referred to as peak-splitting. If reflected spectra containing peak splitting are interpreted using an, e.g., industry standard peak picking algorithm [2,3], then an observed shift between consecutively measured spectral peaks can contain an additional contribution due to switching back and forth between two or more competing peaks. As a consequence of a peak switching incident, the measured signal will contain a jump which offsets all points after the peak switching incident, until the next peak switching incident occurs; effectively adding a distortion to the signal which shows strong similarities with an additive multi-level random telegraph noise signal. The occurrence and severity of peak splitting may depend on, e.g., the fibre deformation state [4,5,6], grating quality [7], as well as a potential deterioration of the grating, or the attachment of the grating [8,9], over time.

The usability of fbg measurement data can be severely limited due to the presence of peak splitting artefacts [10]; this is especially the case for applications which require a high temporal accuracy, such as fatigue monitoring of civil structures [11,12,13], which are extremely sensitive to the presence of peak splitting artefacts. Considering fatigue monitoring as application, any artificial jump(s) in the strain signal(s) will translate to artificial load cycles and therewith affect the calculation of the cumulative fatigue damage over a, e.g., ten-minute measurement interval [14]; as a result, data containing peak splitting can lead to severe over-estimation of the actually accumulated damage.

Peak splitting has the potential to render a long term operational measurement campaign useless when only the peak wavelengths are stored; saving the raw spectral data can result in a significant amount of data which needs to be saved, and is typically only practicable for quasi-static load cases allowing low sample rates, or laboratory experiments at high sample rates with a limited number of experiments [8]. Hence, the presence of peak splitting artefacts in data requires additional post-processing routines, in particular whenever a re-processing of the raw spectral data with peak tracking methods is not possible.

The aim of the post-processing routines presented in this article is to remove spurious jumps, which range from an arbitrary sequence of single impulses to multi level signal offsets spanning several seconds. When considering dynamic signals, typically impulsive distortions, or short duration burst, are considered [15,16,17,18]. When dealing with random telegraph signals (rts), the focus is often on denoising these signals from different noise sources [19,20]. When random telegraph signals are considered as the noise source, the underlying signal is often constant or changes step-wise [21]. The application considered in this article deals with dynamic signals which are corrupted with multi-level random telegraph noise.

In this article, two methods will be presented which can be used to remove peak splitting artefacts from operational fbg (strain) measurements. Both reconstruction methods consist of a two stage approach applied on the sample wise difference signals, which are directly related to the wavelength shifts. The goal of the first stage is to detect difference samples which are attributed to wave-length shifts affected by peak switching, and label those as outliers. The first method detects outliers based on a threshold filtering rule which is derived from the histogram of the sample wise differences; the second method is based on splitting the difference signal into shorter segments, which are subsequently assessed on outliers. During the second stage, the difference samples identified as outliers will be replaced by estimates. To conclude the reconstruction, the cumulative sum of the sample-wise difference signal is computed.

The performance of the reconstruction methods will be verified on FBG strain measurements obtained from an operational offshore wind turbine support structure. The data sets are selected randomly, and are ordered such that they are increasingly difficult to reconstruct. The results will be presented in terms of reconstructed time series. The results show that the proposed methods can be used to successfully reconstruct the dynamic content of the corrupted strain time series. The results also show that a reconstruction of the dynamic part of the signal is possible if the magnitude of the difference samples caused by artificial jumps are larger than the magnitude of the nominal difference samples. Whether the static and/or low frequent band of the measurement data can be reconstructed depends on the severity and amount of peak splitting.

## 2. Peak Splitting Artefacts in Measurement Data

A fibre Bragg grating (fbg) is a periodic modulation of the refractive index along a section of an optical fibre. When a broadband light pulse propagates through a grating, a part of the incident spectrum will be reflected [22]. The reflected narrowbanded spectrum is characterized by a single peak at its central wavelength, also called the Bragg wavelength. As shown in Equation (1), for a uniformly spaced grating, this wavelength (λ) is proportional to the grating period (Λ) and the effective index of refraction ($n_{eff}$); both the effective index of refraction as well as the period of the Bragg grating will be affected by any applied mechanical strain or temperature [4].

(1) $$\lambda =2n_{eff}\Lambda$$

For a fibre connected to a specimen, the temperature compensated, mechanically induced strain ($\epsilon_{m}$) can be expressed as a function of the wavelength (λ) and temperature (*T*), as outlined in Equation (2) [1]; in which κ denotes the gauge factor, $\lambda_{0}$ denotes a reference wavelength, $\alpha_{\delta }$ defines the thermal sensitivity of the index of refraction, whereas $\alpha_{sp}$ and $\alpha_{f}$ denote the thermal expansion coefficient of the specimen and the fibre, respectively, and $T_{0}$ denotes a reference temperature.

(2) $$\epsilon_{m}\left(\lambda ,T\right)=\frac{1}{\kappa }\frac{\lambda -\lambda_{0}}{\lambda_{0}}-\left(\frac{\alpha_{\delta }}{\kappa }+\left(\alpha_{sp}-\alpha_{f}\right)\right)\left(T-T_{0}\right)$$

**Figure 1:**
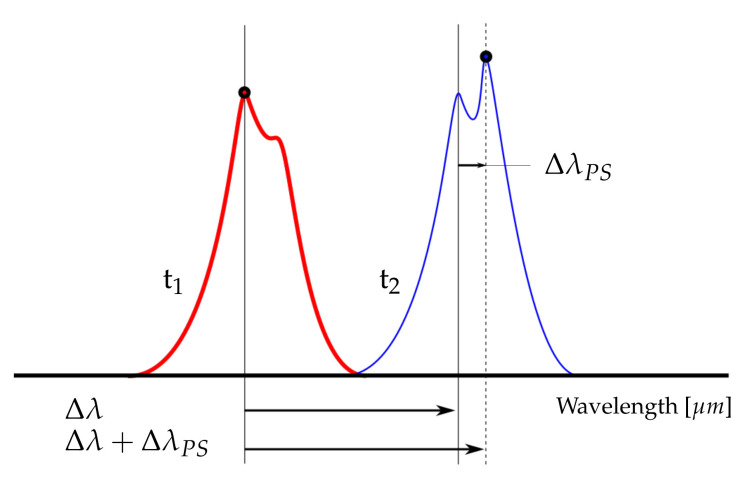
Illustration of peak splitting and switching between two consecutively measured spectra at time instance $t_{1}$ and $t_{2}$, in which Δλ is the nominal shift and $\Delta \lambda_{PS}$ is a contribution due to peak switching.

When the malfunction referred to as peak splitting is present, the reflected spectrum is no longer defined by a single central wavelength, but rather by several wavelengths. As a result, the reflected spectrum will contain multiple peaks around the central wavelength, hence the term peak-splitting. This is illustrated for two idealised, successively measured, reflected spectra in Figure 1. Most interrogators will identify whatever peak is highest and return the corresponding wavelength. If peak switching occurs, i.e., a different peak becomes the highest and gets picked by the interrogator, the measured wavelength shift (Δλ) will now contain an additional contribution ($\Delta \lambda_{PS}$). Similarly, adding the shift due to peak switching to the measured wave length, will result in $\Delta \lambda_{PS}$ as defined in Equation (3); this contribution will directly affect the wavelength dependent part of the mechanical strain as defined in Equation (2), and will result in an instantaneous jump in the strain time series, which is not physically observable.

(3) $$\lambda_{ps}=\lambda +\Delta \lambda_{ps}\;⟶\;\epsilon_{m}=f\left(\lambda_{ps},T\right)$$

**Figure 2:**
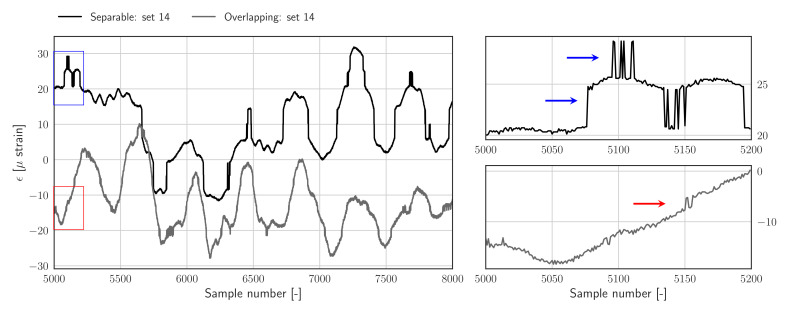
**Left**: two examples of raw strain measurements clearly showing peak switching artefacts as jumps which offset parts of the signal as well as spikes. **Right**: the top and bottom figures show zoomed-in views of the blue and red rectangles, displaying jumps in the separable and overlapping data set, respectively.

To illustrate to effect of peak splitting artefacts two different strain measurements are presented in the left-hand side of Figure 2. The jumps due to peak switching incidents are clearly visible in both signals. Zoomed-in views of both signals, presented in the right-hand side of Figure 2, reveal a strong difference between the amplitudes of the jump with respect to the local variation of the respective strain signal; when looking at the top right figure, large jumps are visible which can be clearly separated from the nominal strain variations, whereas in the lower right figure, the jump amplitudes are closer to the nominal variation of the strain samples.

**Figure 3:**
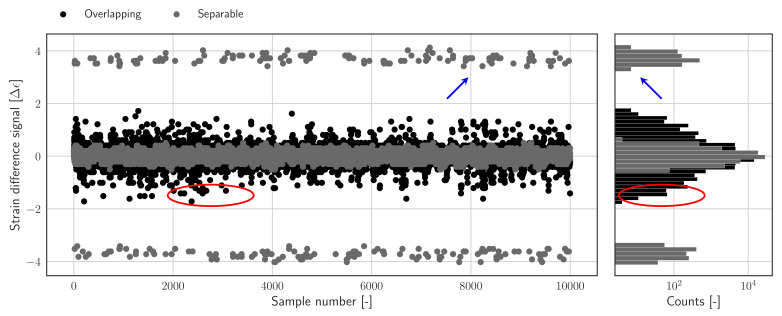
**Left**: Sample wise difference signal, showing peak switching incidents as outliers. **Right**: Histograms of the sample wise difference signals. Indicating the distribution of nominal difference samples and shifted difference samples, which can be overlapping or mutually exclusive (separable). Outlying difference samples in the separable and overlapping data set are indicated by blue and red annotations, respectively.

The difference between the two signals presented in Figure 2 can be clearly illustrated by looking at the corresponding sample wise difference signals, which are shown in Figure 3, and defined in Equation (4); the histograms of the sample wise difference signals are also shown in Figure 3. Both representations clearly indicate the distribution of the nominal difference samples as well as the samples which are shifted due to peak switching; it should be noted that the shifts can result in overlapping or mutually exclusive (separable) clusters of difference samples, which are indicated by blue and red annotations, respectively.

## 3. Reconstruction Methods

The problem of removing jumps from the original strain time series has been transformed into denoising the corresponding difference time series, as defined in Equation (4); in which $\epsilon \in R^{Nx1}$ represents a single, uniformly sampled, strain time series containing *N* samples, $\Delta \epsilon \in R^{Nx1}$ represents the corresponding sample wise difference signal, and $\epsilon_{k}$ and $\Delta \epsilon_{k}$ denote the *k*-th sample taken from the respective time series.

(4) $$\Delta \epsilon_{k}=\left\{\begin{array}{cc} \epsilon_{k+1}-\epsilon_{k}, & 0<k\leq N-1\\ 0, & k=0 \end{array}\right.$$

(5) $${\hat{\epsilon }}_{k}=\sum_{i=0}^{k}\Delta {\hat{\epsilon }}_{i}+\left(b+ka\right)$$

The difference signals are denoised by applying an outlier detection and replacement step. The outlier detection methods which will be used to label the samples in Δϵ as a nominal sample, or outlier, will be presented in Section 3.1. Once the outliers have been detected, each outlier will be replaced by an estimate—the interpolation based approach used in this article will be presented in Section 3.2. After the difference signal has been denoised, the cumulative sum is calculated to obtain the reconstructed strain time series, as defined in Equation (5); in which $\hat{\epsilon }\in R^{Nx1}$ represent the final reconstructed strain time series and $\Delta \hat{\epsilon }\in R^{Nx1}$ represents the corrected sample wise difference signal. Since the mean value is lost in the process, a linear error correction, defined by the coefficients *a* and *b*, can be used which adds a mean value and/or drift correction to the signal. Details on recovering a mean value, as well as the drift correction, are presented in Section 3.3.

### 3.1. Outlier Detection

The objective of the first stage is to detect and label all difference samples which have a component which is related to the wavelength shift caused by peak splitting. Two possible methods for detecting these outliers will be presented in this article. In Section 3.1.1, a threshold filter will be introduced, which is aimed at differentiating between nominal samples and outliers using the histogram of the full signal. In Section 3.1.2, a variant of the Hampel filter will be outlined, which is aimed at detecting outliers in short signal segments using a segment specific threshold.

#### 3.1.1. Histogram Based Filtering Threshold

Equation (6) describes a sample wise outlier detection scheme. The difference sample $\Delta \epsilon_{k}$ is compared to a constant threshold value *S*. If $|\Delta \epsilon_{k}|$ exceeds the value of *S* the sample will be labeled as outlier $\left(l_{k}=1\right)$; if the value of $\Delta \epsilon_{k}$ is in [−S,S], the sample will be labeled as inlier $\left(l_{k}=0\right)$. Repeating this process for all samples will result in the array *l*, which labels all samples as either inlier or outlier.

(6) $$l_{k}=\left\{\begin{array}{cc} 0, & |\Delta \epsilon_{k}|\leq S,\\ 1, & |\Delta \epsilon_{k}|>S. \end{array}\right.$$

An approximate value of *S* can be determined using Algorithm 1. In a nutshell, the algorithm is implemented to find the domain of the nominal samples in the histogram of the difference signal (Figure 3).

**Figure 4:**
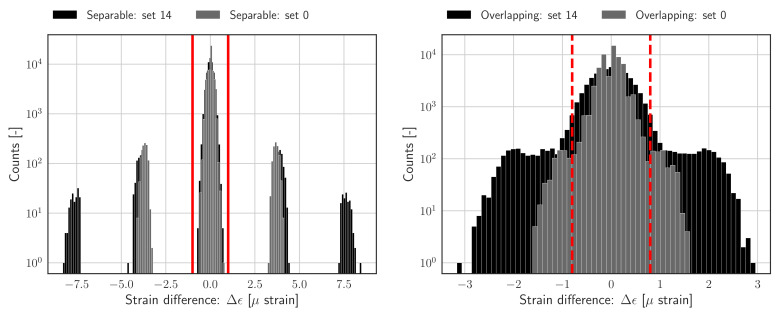
**Left**: separable histograms for two time series, with clear gaps between clusters belonging to nominal and shifted samples, as well as exact domain boundaries (red lines); set 14 contains 4 outlier clusters (black), whereas set 0 contains 2 outlier clusters (grey). **Right**: overlapping histograms for two time series with obvious overlap between clusters, and estimated domain boundaries (red); notable difference between the range and the shape of the histogram belonging to set 14 and set 0.

In case the difference histogram contains separable clusters, as can be seen in Figure 4, the task of determining *S* reduces to detecting discrete gaps in the histogram which separate the nominal difference samples from shifted samples. These gaps define the domain of the nominal difference samples and can be used directly to construct the threshold. The algorithm is therefore configured with a threshold th equal to zero. In case the distributions of nominal and shifted samples do overlap, i.e., when the jumps due to peak-splitting are of the same order of magnitude as the nominal variations in the signal, *S* cannot be derived exactly since the domain of the nominal samples is not defined explicitly; instead, the threshold will be set to a relatively low number of samples, e.g., th=0.01N. Practically speaking, the value of *S* will be derived as the average of the bin centres for which the bin count first drops below th. In other words, the threshold should be chosen such that all shifted samples will be labelled as outliers, while labelling as few as possible nominal samples as outliers.
**Algorithm 1** Histogram based threshold selection.$b_{r}=10$$b_{c},c\leftarrow$hist(Δϵ, nr.bins = $range\left(\Delta \epsilon \right)\times b_{r})$*m**th*:▹Bin resolution [bins/unit]▹Bin centres, bin counts▹Threshold calibration factor▹Threshold on bin counts$$th=\left\{\begin{array}{ll} 0, & \mathrm{non}-\mathrm{overlapping},\\ \approx 0.01\times N, & \mathrm{overlapping}\;\mathrm{distributions}. \end{array}\right.$$$$S=m\times 0.5\times \left(\min\left(b_{c}\left[\left(c\leq th\right)\right]\right)+\max\left(b_{c}\left[\left(c\leq th\right)\right]\right)\right)$$

The threshold value on the histogram count (th), as well as the bin density ($b_{r}$), which is used in creating the histogram, have to be calibrated for each application — and potentially for different loading conditions. The objective is to keep the value of *S* as large as possible; however, if the value of *S* is too large, outliers won’t be detected, whereas an unnecessary reduction of *S* will label more physical correct samples as outliers, and put more emphasis on the outlier replacement methods presented in Section 3.2. The calibration procedure is straightforward, and the speed of the implementations allows to conduct a sensitivity study.

#### 3.1.2. Hampel Identifiers

The Hampel identifier [23] presented in Equation (7), belongs to the class of decision filters, and uses an adaptive threshold to discriminate a sample as in- or outlier. The threshold value for the *k*-th sample, $S_{k}$, is defined as a statistical measure (*f*) of the corresponding sliding window ($W_{k}^{w}$) — as outlined in Equations (8) and (9), respectively. The moving window defined in Equation (9) is typically truncated if the window does overlap with the begin or end of the signal. Note that setting $S_{k}$ to a constant value will result in the threshold based decision filter defined in Equation (6).

(7) $$l_{k}=\left\{\begin{array}{cc} 0, & |\Delta \epsilon_{k}|\leq S_{k},\\ 1, & |\Delta \epsilon_{k}|>S_{k}. \end{array}\right.$$

(8) $$S_{k}=f\left(W_{k}^{w}\right)$$

(9) $$W_{k}^{w}=\left\{\Delta \epsilon_{k-w},\ldots ,\Delta \epsilon_{k},\ldots ,\Delta \epsilon_{k+w}\right\}$$

The Hampel identifier used in this article will follow the more conventional definition and will be defined on non-overlapping signal segments instead of sliding windows. In Equation (10), the sample wise difference signal Δϵ is reshaped into an array holding *n* row-wise segments of length *w*, of which the i-th element will be represented by $R_{i}^{w}$ as defined in Equation (11). Zero padding can be used to maintain consistent segment lengths if n×w≠N.

All samples in a segment are subsequently assessed on being an in- or outlier using a decision rule as defined in Equation (12); in which *i* represents the segment number as function of the sample index *k* and the segment length *w*. The segment wise threshold value, $S_{i}$, presented in Equation (13), is now defined for each segment instead of each sample (sliding window).

(10) $$\Delta \epsilon \in R^{Nx1}\overset{reshape}{\to }\Delta \epsilon^{\prime }\in R^{nxw}$$

(11) $$\begin{array}{c} R_{i}^{w}=\left\{\Delta \epsilon_{i,1}^{\prime },\ldots ,\Delta \epsilon_{i,w}^{\prime }\right\},\;i=1..n \end{array}$$

(12) $$l_{k}=\left\{\begin{array}{cc} 0, & |\Delta \epsilon_{k}|\leq S_{i},\\ 1, & |\Delta \epsilon_{k}|>S_{i}. \end{array}\right.,\;i=\left\lfloor k/w\right\rfloor$$

(13) $$S_{i}=f\left(R_{i}^{w}\right)$$

The segment wise threshold will be defined using an unbiased scale estimate of the standard deviation for Gaussian data which is based on the median absolute deviation (mad), as presented in Equations (14) and (15), respectively. This scale estimate has the desirable characteristic of exhibiting outlier resistance [24,25]. Therefore, it will be used to formulate the segment-wise threshold levels as defined in Equation (16). However, the situation can arise in which more than 50% of the samples are outliers; in that particular case the mad scale estimate for the standard deviation will represent an estimate of the standard deviation of the outliers. As a consequence, $S_{k}$ will be too large, which renders the filter insensitive to outliers, with the consequence that likely all samples in the segment will be labelled as inliers. Hence, these types of filter can degenerate on individual segments containing more than 50% of outliers (or identical samples) [23]. To counter this issue, one can increase the segment length, or apply an additional median filter on the segment-wise threshold values ($S_{k}$). This median filter replaces all values of $S_{k}$ which belong to the segments containing more than 50% of outliers by, e.g., the median of the threshold values contained within the lowest 96^th^ percent of the values of $S_{k}$.

(14) $${\hat{\sigma }}_{i}=1.4826\times {\mathrm{MAD}}_{i}$$

(15) $${\mathrm{MAD}}_{i}=\mathrm{med}(|R_{i}^{w}-\mathrm{med}\left(R_{i}^{w}\right)\left|\right)$$

(16) $$S_{i}=c\times {\hat{\sigma }}_{i}$$

The only required tuning parameters of this method are the length of the segments (*w*: Equation (10)) as well as the threshold scale parameter (*c*: Equation (16)). As outlined, if the segment is too short it is possible that all, or the majority, of the points are outliers, potentially resulting in a degradation of performance on a given number of segments. It is recommended to choose the segment length such that each segment of the raw data can be approximated by a linear model. The threshold scale parameter directly influences all segment thresholds, if this value is too large outliers potentially will remain undetected, whereas a too small number will result in detecting nominal samples as outliers and putting more emphasis on the outlier replacement step. It is therefore suggested to perform a sensitivity study of the reconstruction performance with respect to the segment length as well as the threshold scale parameter.

### 3.2. Outlier Replacement

The second step in the reconstruction process is aimed at replacing all identified outliers in the difference signal by estimates. The outlier replacement step will be explained for the outlier detection method presented in Section 3.1.1. For each outlier which has to be replaced, a buffer containing a number of neighbouring outlier-free difference samples is built which subsequently will be used to determine a replacement via a least squares regression analysis. The buffer, $b\in R^{n_{b}x1}$, as shown in Equation (17), holds $n_{b}$ nominal difference samples which are located at indices *i*. The linear model in Equation (18) will be used to construct a local approximation of the difference samples contained in the buffer. Writing Equation (18) in matrix form for all entries in *b* results in Equation (19); in which $\hat{x}$ represents the vector containing the estimates of model coefficients, and *e* represents the residual vector. Solving the model coefficients $\hat{x}$ using the least squares method, and substituting the estimated coefficients for *a* and *c* in to Equation (18), allows us to construct an outlier replacement at the desired value of *k*. Other interpolation strategies can be found in, e.g., [15].

(17) $$b=\left[\begin{array}{c} \Delta \epsilon_{i_{1}}\\ \vdots \\ \Delta \epsilon_{i_{n_{b}}} \end{array}\right]$$

(18) $$\Delta \epsilon_{i}=ai+c$$

(19) $$b=\left[\begin{array}{cc} i_{1} & 1\\ \vdots & \vdots \\ i_{n_{b}} & 1 \end{array}\right]\left[\begin{array}{c} \hat{a}\\ \hat{c} \end{array}\right]+e=H\hat{x}+e$$

(20) $$\hat{x}={\left(H^{T}H\right)}^{-1}H^{T}b$$

The concepts explained in this section can be easily extended to the Hampel filter based outlier detection method presented in Section 3.1.2, with the only difference that the notion of an outlier specific buffer will now be replaced by a segment specific buffer, which may contain several outliers. As a consequence, the length of the segment/buffer will not equal the amount of samples which is effectively used to build the local approximation, instead the latter will always be based on less samples.

**Figure 5:**
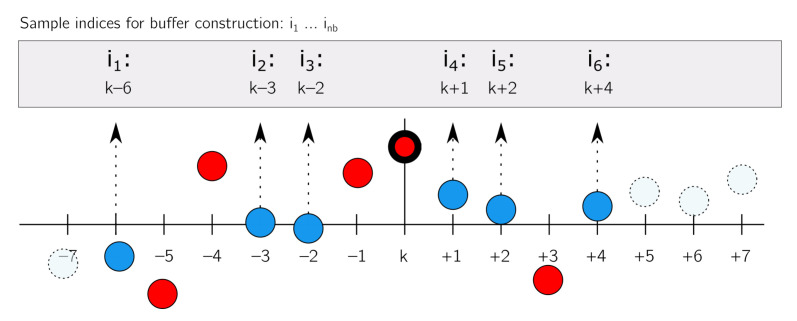
Illustration of the buffer construction (sample selection) with $n_{b}=6$ samples for an outlier at index *k*, and the effect of outliers on the effective buffer length. Red circles indicate outliers, which will not be included in the buffer; blue circles indicate nominal difference samples and dashed circles indicate samples outside the buffer.

Fitting a linear model is suggested if the shortest relevant period in the signal is larger than the effective length of the buffer. It should be emphasised that the effective length of the buffer can exceed $n_{b}$ due to the potential presence of outliers in the direct vicinity of the target outlier, as illustrated in Figure 5.

### 3.3. Integration and Drift Correction

After having denoised the difference data, the cumulative sum is used to reconstruct the strain time signal. A problem arising due to taking the cumulative sum is that errors in the outlier replacement step can accumulate and cause a drift in the reconstruction. This drift can be assessed visually once the reconstruction has been performed for the considered signals.

**Figure 6:**
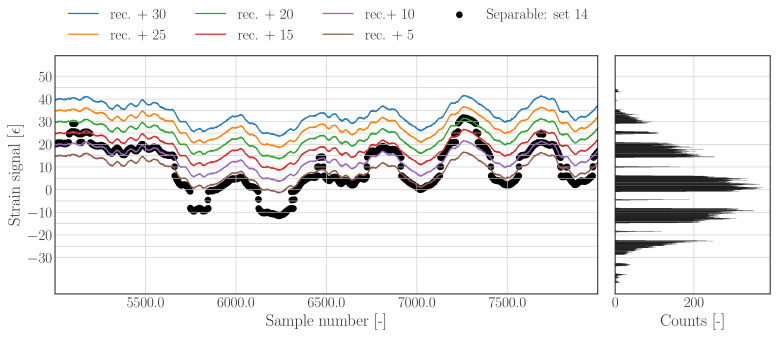
**Left**: subset of the raw and reconstructed time series with different mean values, illustrating the problem of deriving a mean value from the raw signal. **Right**: histogram of the full raw signal illustrating the distribution of samples over the signals range as well as the jumps in the data.

A practical problem associated with speak splitting is the ambiguity of the signal’s mean value. The effect of peak splitting on the mean value does not have to be zero, even though the number and duration of upward jumps and downward jumps are exactly equal. Furthermore, from the raw signal it cannot be derived with certainty which realisation of the reconstruction corresponds to the nominal state—as illustrated in Figure 6, which shows the corrupted raw data, as well as six reconstructions with different mean values. This figure illustrates that each of the reconstructions does overlap with a part of the raw signal. Assessing the histogram of the raw signal in Figure 6 (right) shows that most of the samples fall between 8–16 micro strains, hence shifting the reconstruction by 20 micro strain would result in a maximization of the overlap between the reconstruction and raw signal.

**Figure 7:**
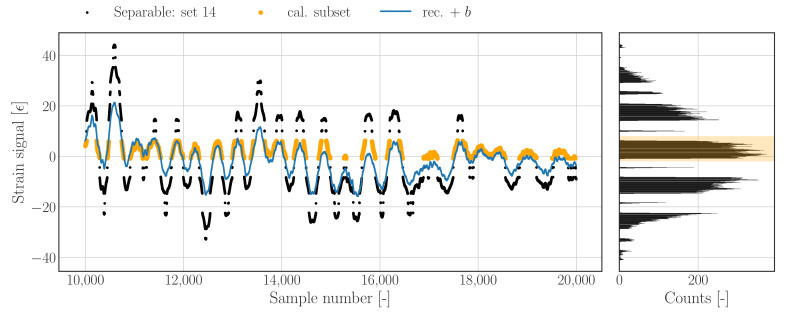
Raw (union of black and orange) and reconstructed time series (blue) with calibrated mean value based on optimizing the overlap of the reconstruction with a calibration subset (orange).

A pragmatic solution for determining a mean, as well as drift correction, can be obtained by solving the optimization problem presented in Equation (21). To achieve the latter, the 2-norm of the difference between the raw data ($\epsilon_{s}$), and the reconstructed data (${\hat{\epsilon }}_{s}$) with an additional linear trend, is minimized for the parameters of that linear model; in which *x* equals the vector containing integer sample numbers belonging to all values of ϵ which fall between a lower and upper strain bound, $\epsilon_{lb}$ and $\epsilon_{ub}$, respectively. The offset term (*b*) has to shift the reconstruction to an appropriate mean level, whereas the trend (ax) has to account for a potential linear drift in the reconstruction. Applying bounds to the calibration subset is not necessary in every situation, but can be used to define the region of the raw data which is used to maximize the overlap, and therewith effectively avoid potential local optima. Figure 7 shows the results of a drift correction with explicit bounds on the calibration subset. The bounds are selected such that the reconstruction will find an optimal offset which maximizes the overlap with the majority of the raw signal samples as indicated by the orange samples/band.

(21) $$\begin{array}{cccc} \; & \underset{a,b}{\mathrm{minimize}}\; & \; & {∥\epsilon_{s}\left[x\right]-\left({\hat{\epsilon }}_{s}\left[x\right]+ax+b\right)∥}_{2}\\ \; & \mathrm{with}:\; & \; & \left\{x\in Z\;|\;\epsilon \left[x\right]\in \left[\epsilon_{lb},\epsilon_{ub}\right]\right\} \end{array}$$

**Figure 8:**
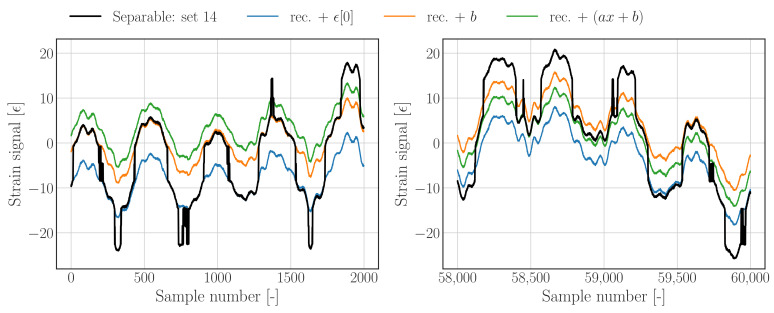
Raw and reconstructed time series with calibrated mean value using all samples as calibration subset.

The results in Figure 8 have been generated without explicit strain bounds, hence all samples are used in solving the optimization problem. Besides showing results for an offset (orange) and a trend optimization (green), this figure also illustrates a reconstruction whose offset is set to the first value of the raw data (blue).

Figure 8 (left) shows the results at the beginning of the data set, whereas the right figure depicts the results towards the end of the data set; it has to be noted that the realisation which is based on an offset equal to the first sample of the raw signal, is random and could be related to any state as illustrated in Figure 6, whereas the offset and trend optimized realisations are based on minimizing (the overall) the distance between the raw data and the reconstruction.

Considering most practical applications, it is not necessary to use the full bandwidth of the signal. For those cases, it might not be necessary to use the drift correction as proposed above, instead band-pass or high-pass filtered versions of the reconstructed data can be used directly on the reconstructions. The latter filtering will remove the mean value as well as most of the accumulated drift. In order to ensure that this approach is valid, hence only the mean and the low frequent drift has been removed, it is recommended to compare the dynamic response of the filtered reconstruction and the raw data on overlapping subsets.

The reconstructions presented in the remainder of this article are not mean and/or drift corrected, nor filtered, in order to illustrate the quality of the raw reconstructions.

## 4. Data Description

The FBG strain measurements used in this paper have been obtained from an operational offshore wind turbine support structure. The measurement data has been selected randomly over a period of 1 month. Subsequently two data sets containing 15 signals each have been selected: one containing overlapping difference histograms (*Overlapping.hdf5*), and the other containing separable histograms (*Separable.hdf5*) — as introduced in Figure 4. The signals in both data set have been sorted on the range of the individual difference signals — The latter can be used as a simple measure for detecting the presence of peak splitting in the data. An overview of the included data is presented in Table 1.

The data and software which are used for this article are made available as python package (py_peak_splitting, codebase: github.com/OWI-Lab/py_peak_splitting, accessed on 12 October 2021) [26] which is hosted on Github under a CC BY-NC-SA 4.0 License. In addition to real-world measurement data, this python package does include demonstrations, as well as a module to simulate harmonic signals containing different types of peak splitting alike artefacts

## 5. Results

In this section, the results for the threshold-based reconstruction method as well as the Hampel filter based reconstruction method will be presented. It is chosen to perform a qualitative assessment of the reconstruction accuracy, since no peak splitting free counterparts to the presented signals are available—the latter would allow to perform a straightforward deterministic assessment of the reconstruction results. In order to assess and compare the reconstruction methods qualitatively, the same data subsets will be used for visual assessments of the reconstruction accuracy. Section 5.1 will compare the results of both methods for the signals contained in *Separable.hdf5*, and Section 5.2 will present the results for the signals contained in *Overlapping.hdf5*. The data sets will be processed with the default parameters presented in Table 2. To conclude this chapter, Section 5.3 will present a brief sensitivity study on the threshold parameter settings for both methods for the case of an overlapping difference histogram.

### 5.1. Separable Difference Histograms

**Figure 9:**
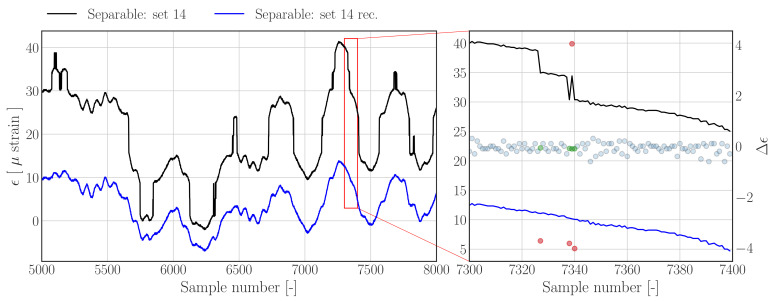
**Left**: Subset of data set 14 from *Separable.hdf5* (dashed), and reconstructions (solid) using the *threshold filter based method*. **Right**: Zoom in on region containing jumps. Nominal difference samples are indicated with blue dots, outliers and corresponding outlier replacements are indicated by red and green dots, respectively.

Figure 9 shows the reconstruction results for a separable data set obtained using the threshold filter-based reconstruction approach outlined in Section 3.1.1—see Figure 4 for the corresponding raw histogram. The raw signal shows evident jumps, which become more evident when zooming in on a smaller subset as shown in Figure 9 (right); the latter also shows the nominal difference samples, which are indicated by blue dots, as well as the outliers and corresponding outlier replacements, which are indicated by red and green dots, respectively. The results show that all outlying difference samples are detected, and that the reconstructed strain time series has been denoised effectively.

**Figure 10:**
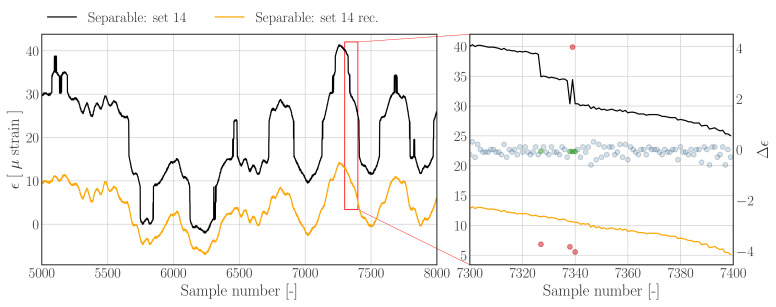
**Left**: Subset of data set 14 from *Separable.hdf5* (dashed), and reconstructions (solid) using the *Hampel filter based method*. **Right**: Zoom in on region containing jumps. Nominal difference samples are indicated with blue dots, outliers and corresponding outlier replacements are indicated by red and green dots, respectively.

The results in Figure 10 are obtained by using the Hampel filter (Section 3.1.2) as the outlier detection method in combination with the outlier replacement method outlined in Section 3.2. The reconstructed time series in Figure 10 shows that the jumps have been removed effectively.

**Figure 11:**
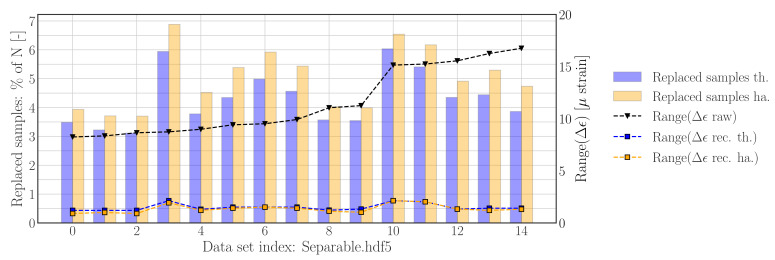
Reconstruction results using *threshold filter based method* and *Hampel filter based method* with default settings for data contained in *Separable.hdf5*. **Left axis**: number of replaced samples per file. **Right axis**: range of sample-wise difference signal before (raw) and after reconstruction (rec).

The reconstruction processes, used to generate the results for Figure 9 and Figure 10 have been repeated on all data sets contained in *Separable.hdf5*. The results, in terms of the percentage of replaced samples, as well the range of the raw and reconstructed strain difference time series, are presented in Figure 11. These results for the threshold based reconstruction approach show that 3–6% of the samples have been replaced, and that the range of difference data has been reduced by a factor of ∼5. Data set 3, 10 and 11 do deviate slightly in terms of the range associated with the nominal difference samples—as can be seen in Figure A2 and Figure A3—which explains the perceived lower range reduction. The results obtained with the Hampel filter based approach show that the amount of replaced samples as well as the reduction of the range of the reconstructed difference signals is similar to the results which have been obtained for the threshold filter based reconstruction method.

The spread in the replaced difference samples in the right graph of Figure 9, as well as Figure 10, is significantly lower than the spread in the nominal samples—this observation generalizes to all other data sets which are presented. Part of this observation is rooted in the noisy nature of the measurement data itself, which can mask the underlying difference signal and reduce the accuracy of the interpolation based replacement approach. As stated in Section 3.2, the length of the window which is used to create the replacements does have an influence as well: Too short window/segment lengths (a few samples) can result in extremely noise sensitive local approximations, potentially resulting in very large reconstruction errors; adequate window/segment lengths in combination with low signal to noise ratios can result in the limiting case that the mean value will be used as replacement and that the information on the ratio diminishes relative to the noise free case; whereas replacements generated using excessively long windows/segments can result in including non-local trends.

### 5.2. Overlapping Difference Histograms

**Figure 12:**
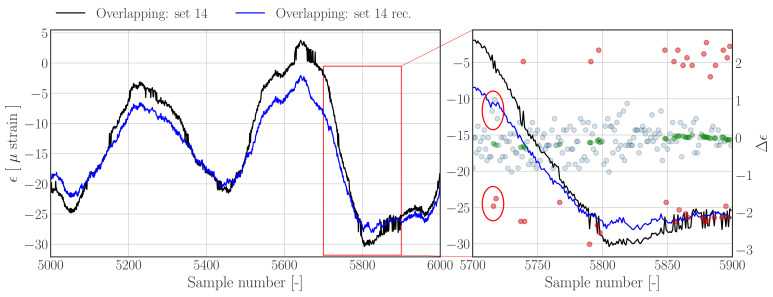
**Left**: Subset of data set 14 from *Overlapping.hdf5* (dashed), and reconstructions (solid) using the *Threshold filter based method*. **Right**: Zoom in on region containing jumps. Nominal difference samples are indicated with blue dots, outliers and corresponding outlier replacements are indicated by red and green dots, respectively.

Figure 12 shows the reconstruction results for an overlapping data set obtained using the threshold filter based reconstruction approach outlined in Section 3.1.1—see Figure 4 for the corresponding raw histogram. In contrast to Figure 9 and Figure 10, the jumps are less evident and closer to the nominal difference samples, as can be seen in Figure 12 (right). The results show that the reconstructed strain time series appear to show less peak splitting artefacts (left), and that outlying difference samples are detected (right). However, a few errors can be noted, for example, between sample number 5715 and 5725 (indicated by the red annotations) it appears that the upward jumps corresponding the downward jumps are not labelled as outliers, resulting in a slight upward bump since the negative difference samples have been detected and replaced.

**Figure 13:**
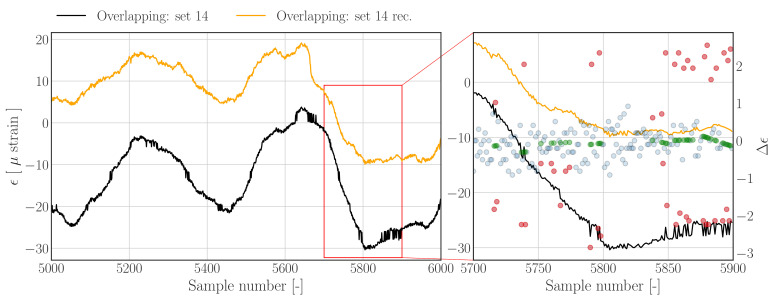
**Left**: Subset of data set 14 from *Overlapping.hdf5* (dashed), and reconstructions (solid) using the *Hampel filter based method*. The segment wise threshold levels are processed using an additional median filter. **Right**: Zoom in on region containing jumps. Nominal difference samples are indicated with blue dots, outliers and corresponding outlier replacements are indicated by red and green dots, respectively.

The reconstruction results using the Hampel filter based method are presented in Figure 13; comparing the latter figure to the results obtained with the threshold based approach, as presented in Figure 12, shows that both methods produce different results. Although jumps are clearly removed from the signal, it can be concluded that the reconstruction does not track the raw signal as well as the threshold based approach. Furthermore the signal appears to have a slightly compressed range, indicating that perhaps too many outliers have been selected. The differences between both approaches can be brought back to the segment wise threshold values which are defined using the scale estimate of the standard deviation per segment, as defined in Equation (15). Furthermore, the segment wise sample replacement can affect the results in case the buffer contains a high number of outliers, which will reduce the number of nominal samples which are used to construct the outlier replacements.

**Figure 14:**
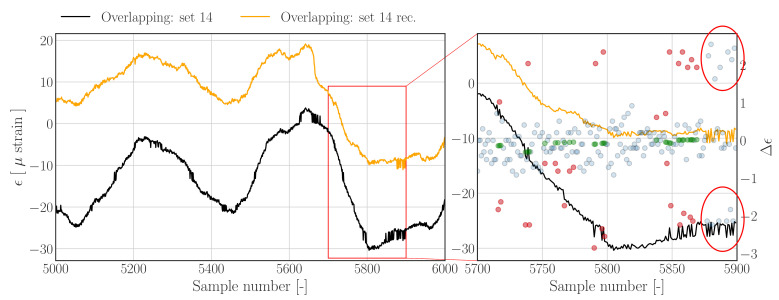
**Left**: Reconstruction results as presented in Figure 13 without applying a median filter on the segment wise threshold levels. **Right**: The last 25 samples contain more than 12 outliers, effectively resulting in a threshold level which will be leave all outliers undetected—as indicated by the red annotations.

In order to generate the results in Figure 13, the segment wise threshold levels defined in Equation (16) are processed using an additional median filter—as outlined in Section 3.1.2. In case this additional filtering is omitted, the results in Figure 14 are obtained. The red annotation in this figure highlights undetected outliers, which fall onto a segment consisting of more than 50% of outliers; the latter causes the segment specific MAD to increase significantly and therewith rendering the Hampel identifier insensitive to outliers.

**Figure 15:**
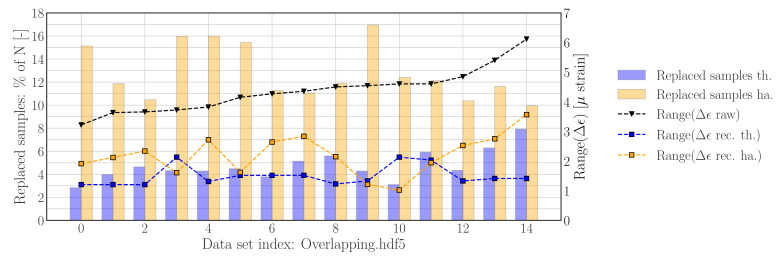
Reconstruction results using *threshold filter-based method* and *Hampel filter-based method* with default settings for data contained in *Overlapping.hdf5*. **Left axis**: number of replaced samples per file. **Right axis**: range of sample-wise difference signal before (raw) and after reconstruction (rec).

**Figure A1:**
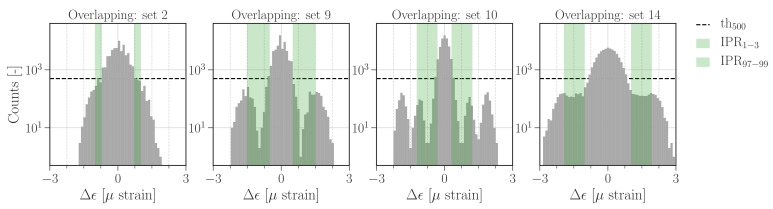
Histogram of sample wise strain difference signals for four overlapping cases, including inter percentile ranges (IPR).

**Figure A2:**
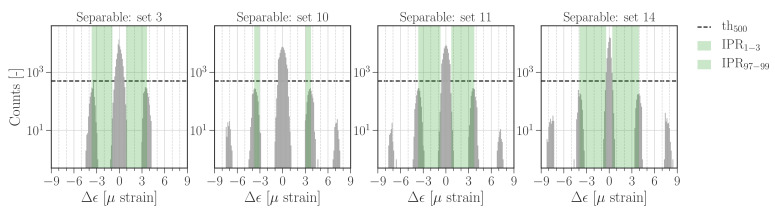
Histogram of sample wise strain difference signals for four separable cases, including inter percentile ranges (IPR).

**Figure A3:**
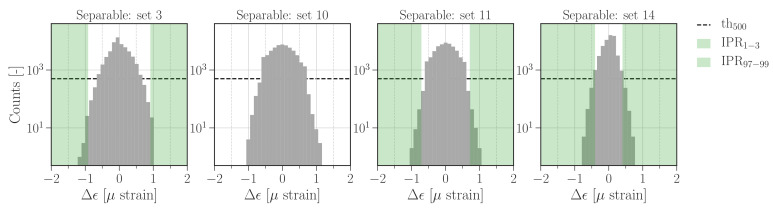
Zoom in on histogram of sample wise strain difference signals for four separable cases, including inter percentile ranges (IPR). The distribution of the nominal samples of set 14 is representative for the other data sets which which are not shown in this figure.

The threshold filter-based reconstruction process used to generate the results for Figure 12 has been repeated on the data contained in *Overlapping.hdf5*. The results are presented in Figure 15 and show that 2.8–7.9% of the samples have been replaced. Furthermore, the range of difference data has been reduced by a factor of ∼2.5. The histograms of the difference signals from data set 9 and 10, as shown in Figure A1, deviate from the other data sets; these sets exhibit clear gaps between the distributions belonging to the nominal samples as well as the shifted samples — however, in contrast to the separable case, these gaps are not defined by intermediate bin counts with a value of zero. As a result of having a more clear separation between nominal and shifted samples, the threshold found with Algorithm () will be lower, resulting in a lower range of the reconstructed difference signal. It should be noted that this overlap can potentially be eliminated by increasing the bin density, allowing us to use the approach defined for separable histograms; however, this comes at the risk of introducing more gaps in the nominal distribution, which can be related to, e.g., resolution limits/quantization effects.

The Hampel filter-based reconstruction process used to generate the results for Figure 13 has been repeated on all data sets contained in *Overlapping.hdf5*. Comparing the reconstruction results presented in Figure 15 show that significantly more samples have been replaced by using the Hampel filter based reconstruction method, which also suggests that the threshold settings could be increased to improve performance.

### 5.3. Threshold Sensitivity for Overlapping Difference Histograms

**Figure 16:**
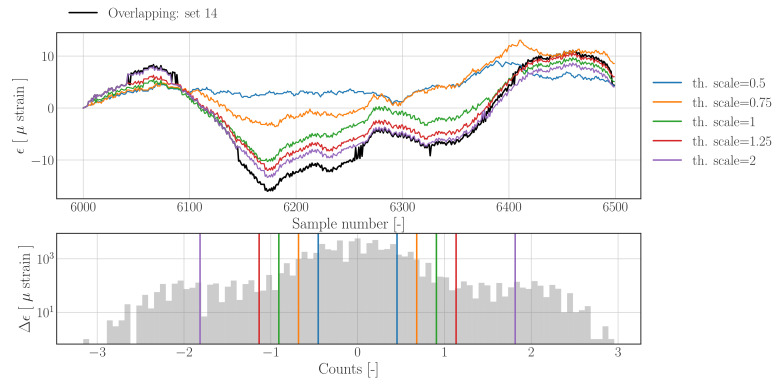
Sensitivity study on reconstruction results using *threshold filter based method* with default settings for data contained in *Overlapping.hdf5*. **Upper**: reconstructed and raw strain time series; first sample of subset has been re-centred at zero to more clearly show the effect of drift. **Lower**: Histogram of the raw difference signal with vertical lines indicating the selected threshold levels which are used for the reconstruction.

The influence of the threshold amplitude on the reconstruction result generated with the threshold filter based method are shown in Figure 16; the reconstructed time series for different threshold values are presented in the upper figure, whereas the raw difference histogram as well as the derived threshold values are shown in the lower figure.

The results clearly show that reducing the threshold level too much will remove physically important information which is lost during the replacement step; this can be seen for the reconstruction using th.scale=0.5, which completely loses track of the raw signal between sample number 6100 and 6300. Increasing the threshold too much, as for th.scale=2, will render the outlier detection insensitive to some jumps, as can be seen between sample number 6045 and 6085, where the purple signal does follow the raw signal (black) during an upward jump. The reconstruction created with th.scale=1.25 shows slightly improved tracking of the quasi-static contributions of the raw signal in comparison with the default reconstruction. When only the dynamic part of the signal is of interest, the signals can be high- or band-pass filtered, which will reduce the difference between both signals significantly.

**Figure 17:**
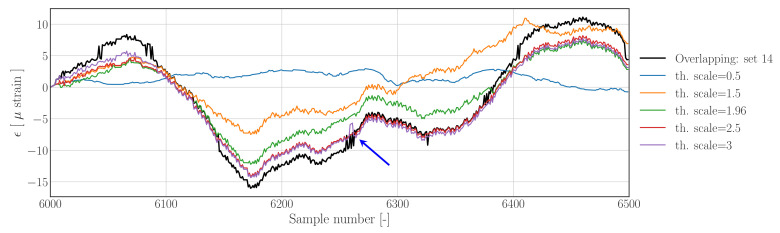
Raw strain time series (black) and *Hampel filter-based* reconstructions at different levels of the threshold scale parameter. The lower the scale parameter the more samples will be detected as outliers, hence the more emphasis is put on the outlier reconstruction method.

Looking at the sensitivity of the reconstruction results generated with the Hampel filter based method in Figure 17, confirms a similar finding; setting the threshold scale parameter to low will result in significant loss of information, a scale parameter of th.scale=2.5 does improve the results, whereas increasing this factor to th.scale=3 would lead to the reintroduction of small jumps as can be seen around sample 6260 (indicated by the blue arrow). The results show that the jumps can been removed effectively provided the threshold scale parameter has been appropriately calibrated.

## 6. Conclusions

As demonstrated in this article, the proposed methods can effectively remove peak splitting artefacts from severely damaged data. Considering practical applications which are focused on the dynamic part of the measurement data, the results show that the effects of peak splitting artefacts can be reduced to a degree where the measurement data is usable for further processing and research applications. However, the reconstruction process is insensitive to the signals original mean value and can add a drift to the reconstruction. Both effects can be reduced by adding an linear trend to the reconstruction whose parameters are optimized such that the overlap between the reconstruction and the raw data is maximized.

Both methods are effective in case the jumps due to peak switching are significantly larger than the strain difference values measured due to the response of the system. The reason for this is that the outlier detection is fairly straightforward in this case. Small differences between both methods can arise due to the outlier replacement step, which use slightly different data subsets to perform the replacements using interpolation. For the threshold filter-based method, a buffer is used which has a fixed length containing the nearest nominal difference samples, whereas the Hampel filter based approach uses a segment of a fixed length, which can contain multiple outliers, which can reduce the number of samples which are available to estimate the replacements.

In case the jumps due to peak switching are closer, or overlap, with the nominal strain differences, the methods do show slight differences. Qualitative results indicate that the threshold filter based method slightly outperforms the Hampel filter-based method. However, the results of the threshold sensitivity assessment show that both methods can remove jumps from the data when parameters are appropriately calibrated. When using the Hampel filter based method, it is advised to apply an additional median filter on the derived segment thresholds in order to filter out thresholds which are derived from segments.

## Figures and Tables

**Table 1:** Overview of available signals and signal properties.

Data Set	Nr. Signals	Nr. Samples (N)	Range of Histogram Widths	Range of OTNR’s *
*Separable.hdf5*	15	60,000	8.25–16.76	0.032–0.072
*Overlapping.hdf5*	15	60,000	3.22–6.12	0.028–0.079

* OTNR: estimate of the ratio between detected outliers and nominal samples.

**Table 2:** Default reconstruction settings.

Separable difference histogram: *Separable.hdf5*				
Method	th.	th. scale	buffer/segment	fit order
th	0	1	25	1
ha	-	3	25	1
Overlapping difference histograms: *Overlapping.hdf5*				
Method	th.	th. scale	buffer/segment	fit order
th	0.01N	1	25	1
ha	-	1.96	25	1

th: Threshold based reconstruction, ha: Hampel filter based reconstruction.

## Data Availability

The data and software that support the findings of this article are made available as python package (py_peak_splitting, codebase: github.com/OWI-Lab/py_peak_splitting, accessed on 12 October 2021) [26] which is hosted on Github under a CC BY-NC-SA 4.0 License.

## Abbreviations

The following abbreviations are used in this manuscript:

fbg	Fibre Bragg grating
med	median
rtn	Random telegraph noise
mad	Median absolute deviation
ipr	Inter percentile range

## Appendix A. Selected Histograms

Figure A1 displays a number of histograms which have been selected from the *Overlapping.hdf5* data set. The histograms displayed in this section are chosen such that they represent the complete data set in terms of the spread in histogram shapes. The histograms corresponding to set 2 and 14 match the image sketched of an overlapping histogram—see Figure 4 for comparison. In contrast to the latter histograms, the ones created for data set 9 and 10 show nearly separated clusters. All histograms have been determined using the default bin resolution setting, which has been set to: 0.1μ strain.

Figure A2 shows some representative difference histograms from data contained in *Separable.hdf5* and Figure A3 shows the corresponding zoom in around the nominal cluster of difference samples. It can be seen that the nominal clusters from data set 3, 10 and 11 are relatively wider than data set 14. Furthermore, it can be seen that data set 10 has no difference samples which belong the the smallest three percent of samples in its nominal cluster.
